# Severe HBV-Associated Hepatitis in Infants from the Same Family: Two Clinical Cases with Different Outcomes

**DOI:** 10.3390/pathogens15080804

**Published:** 2026-07-30

**Authors:** Petar Vasilev, Zhelyazko Badarov, Angel Todev, Petya Argirova, Velina Stoeva, Boriana Chopova, Maria Atanasova, Ivan Baltadzhiev, Mariyana Stoycheva-Vartigova

**Affiliations:** 1 Department of Infectious Diseases, Parasitology and Tropical Medicine, Faculty of Medicine, Medical University, 4002 Plovdiv, Bulgariapetya.argirova@phd.mu-plovdiv.bg (P.A.); ivan.baltadzhiev@mu-plovdiv.bg (I.B.); 2 Clinic of Infectious Diseases, University Multipurpose Hospital for Active Treatment “Sveti Georgi”, 4002 Plovdiv, Bulgaria; 3 Center for Emergency Medical Care, 4002 Plovdiv, Bulgaria; 4 Department of Infectious Diseases, Pulmed University Hospital, 4002 Plovdiv, Bulgaria; 5 Section of Epidemiology, Department of Epidemiology and Disaster Medicine, Faculty of Public Health, Medical University, 4002 Plovdiv, Bulgaria; velina.stoeva@mu-plovdiv.bg; 6 Faculty of Medicine, Medical University, 4002 Plovdiv, Bulgaria; 7 Laboratory of Virology, University Multipurpose Hospital for Active Treatment “Sveti Georgi”, 4002 Plovdiv, Bulgaria; mariya.atanasova@mu-plovdiv.bg; 8 Department of Medical Microbiology and Immunology “Prof. Dr. Elisei Yanev”, Faculty of Medicine, Medical University, 4002 Plovdiv, Bulgaria; 9 Research Institute, Medical University, 4002 Plovdiv, Bulgaria; mariyana.stoicheva@mu-plovdiv.bg

**Keywords:** hepatitis B virus, infants, HBV-associated hepatitis, pediatric acute liver failure, hepatitis D virus, household transmission, immunoprophylaxis

## Abstract

Acute hepatitis B virus (HBV) infection is rare during infancy, and severe symptomatic HBV-associated hepatitis is even less common. We describe two siblings who developed severe HBV-associated liver injury during infancy, two years apart, but experienced markedly divergent clinical outcomes. Clinical, laboratory, virological, microbiological, imaging, therapeutic, and follow-up data were retrospectively reviewed. Household members were subsequently tested for HBV and HDV. The first infant, a 5-month-old girl, presented with HBsAg positivity, negative serological markers for HAV, HCV, and HEV, and severe acute liver injury with a fulminant clinical course that resulted in death. Because anti-HBc IgM, HBV DNA, repeat HBsAg testing, and follow-up serology were unavailable, acute HBV infection could not be confirmed, and the episode was therefore classified as presumed HBV-associated fulminant hepatitis. The rapidly fatal clinical course precluded a comprehensive etiological evaluation, and the available medical records did not fully document the diagnostic work-up. The second patient, a 4-month-old boy, presented with serological and molecular evidence of acute HBV infection, including low-level HBV DNA, total anti-HDV positivity of uncertain clinical significance, and positive CMV IgM/IgG serology. He also had a urinary tract infection (UTI) caused by extended-spectrum β-lactamase (ESBL)-producing *Escherichia coli*. Without HDV RNA and CMV DNA testing, active HDV infection and clinically significant CMV disease could not be definitively confirmed, while passive transfer of maternal anti-HDV antibodies could not be excluded. The patient required prolonged hospitalization, during which he received supportive, replacement, and antimicrobial therapy. He recovered, and long-term follow-up demonstrated HBsAg clearance with undetectable HBV DNA. Testing of household members revealed chronic HBV infection in the mother and maternal grandmother, both of whom had detectable HDV RNA, consistent with ongoing HBV/HDV circulation within the family. However, without viral sequence data from the infants, the source, route, timing, and direction of transmission could not be established. These cases illustrate the potential severity of HBV-associated hepatitis during infancy and its highly variable clinical outcomes. They also underscore the importance of antenatal HBV screening, timely immunoprophylaxis, and cautious interpretation of suspected viral coinfections when molecular confirmation is unavailable.

## 1. Introduction

Hepatitis B virus (HBV) infection remains a major global health concern despite the widespread implementation of effective vaccination programs. According to the World Health Organization, approximately 296 million people are living with chronic HBV infection, with a substantial proportion of the global disease burden attributable to infections acquired early in life [1,2]. Infection during infancy and early childhood is of particular significance due to the high likelihood of progression to chronic infection—reaching up to 90% among infants born to HBeAg-positive mothers—as well as the subsequent risk of long-term complications, including cirrhosis and hepatocellular carcinoma [3,4]. HBV also continues to pose an important public health challenge in Bulgaria. The draft National Programme for Prevention of Viral Hepatitis in the Republic of Bulgaria 2026–2030 calls for improved early diagnosis, sustained high hepatitis B vaccination coverage, HBsAg screening during pregnancy, timely administration of the HBV vaccine at birth, and prevention of vertical and intrafamilial transmission.

HBV infection during infancy has distinct biological and clinical characteristics. Biologically, the immature and relatively tolerant immune response favors viral persistence and progression to chronic infection rather than severe immune-mediated hepatocellular injury [3,4,5]. Clinically, affected infants are usually asymptomatic or have only mild symptoms, and infection is often recognized later through persistent HBsAg positivity. Severe acute hepatitis with marked cytolysis, coagulopathy, and progression to acute liver failure is rarely observed [6,7]. A fulminant presentation contrasts sharply with the expected natural history of early-life HBV infection, making such cases clinically and epidemiologically important.

During infancy and early childhood, HBV is acquired primarily through vertical mother-to-child transmission or early horizontal intrafamilial spread [8]. Consequently, HBV infection identified during this period should be interpreted in the context of several possible scenarios, including perinatal infection, early horizontal household transmission, acute symptomatic infection, and chronic HBV infection first diagnosed during infancy. HBsAg positivity alone does not establish the timing, direction, or mechanism of transmission and should be interpreted in conjunction with serological, molecular, epidemiological, and vaccination history data. Prevention of mother-to-child transmission remains a cornerstone of HBV control strategies [8,9]. Timely administration of the HBV vaccine at birth and, in high-risk settings, combined passive–active immunoprophylaxis with hepatitis B immunoglobulin (HBIG) significantly reduce the risk of perinatal transmission [10,11]. Nevertheless, these transmission routes remain clinically relevant, particularly in settings characterized by gaps in antenatal screening, suboptimal immunization coverage, and insufficient follow-up of at-risk infants [3,9].

In most pediatric cases, HBV infection is asymptomatic or presents with mild clinical manifestations. However, in a subset of cases—particularly during infancy—the infection may follow a severe course, with a fulminant presentation and progression to acute liver failure [3,4,6]. Earlier clinical series have documented fulminant hepatitis B in infants aged 2–5 months, which may be associated with profound coagulopathy and high mortality [6]. Recovery following fulminant disease has also been reported, including in a 4-month-old infant successfully treated without liver transplantation [7]. This marked clinical variability highlights the need for careful evaluation of viral, host, diagnostic, and clinical management factors, particularly when molecular, immunological, and histopathological data are limited.

Coinfection with hepatitis D virus (HDV) warrants particular consideration, as it is associated with increased disease severity and poorer liver-related outcomes in individuals with HBV infection [4]. Notably, several publications suggest that the severity and adverse outcomes of HBV infection in infancy cannot always be explained by specific viral variants or by the infecting dose, highlighting the important role of host-related factors [5,12]. However, data on the role of coinfections and other determinants of clinical variability in acute HBV infection in infants remain limited.

This report describes two siblings who each developed severe HBV-associated hepatitis during infancy, two years apart, but experienced markedly divergent clinical outcomes. The side-by-side analysis of these two cases within a documented household HBV/HDV cluster provides insight into unresolved transmission pathways, missed opportunities for prevention, diagnostic and therapeutic considerations, and the limitations of establishing causality in the absence of viral sequence or immunological data from the affected infants.

## 2. Materials and Methods

This case report presents a retrospective analysis of two infants with severe HBV-associated hepatitis who were hospitalized at the Clinic of Infectious Diseases, University Hospital “St. George”, Plovdiv. Clinical, laboratory, virological, microbiological, imaging, therapeutic, and follow-up data were obtained from the available medical records.

Pediatric acute liver failure was classified according to established diagnostic criteria. It was defined as acute liver injury in a child without known pre-existing chronic liver disease and coagulopathy not corrected by vitamin K administration. The diagnosis was established when INR was ≥1.5 in the presence of clinical evidence of encephalopathy or ≥2.0 regardless of the presence of encephalopathy [13]. Because both patients were infants, encephalopathy was assessed retrospectively based on documented changes in consciousness and neurological status, including marked somnolence, lethargy, impaired responsiveness, progressive depression of consciousness, or hepatic coma. Information on vitamin K administration, plasma replacement, and serial changes in coagulation parameters was extracted from the medical records.

Serological and molecular investigations were performed according to the availability of clinical specimens, the diagnostic capabilities of the hospital laboratory, the urgency of each patient’s clinical condition, and the duration of hospitalization. Specimens for the initial diagnostic evaluation were collected immediately upon admission to the Clinic of Infectious Diseases.

The girl was admitted to University Hospital “St. George”, Plovdiv, on 30 April 2021, approximately two days after symptom onset according to the medical records. Owing to the rapidly fulminant course and fatal outcome, virological testing was limited to basic viral hepatitis markers, including anti-HAV IgM, HBsAg, anti-HCV/HCV Ag/Ab, and anti-HEV IgM/IgG, all performed by ELISA. The virological report was issued on 10 May 2021, one week after the child’s death on 3 May 2021. This period coincided with the COVID-19 pandemic, when the hospital laboratory was operating under a substantial workload, providing important context for the delayed etiological confirmation. Because of the brief hospitalization and the availability of the HBsAg result only after the fatal outcome, extended HBV serology, including anti-HBc IgM, as well as HBV DNA, HDV serology, HDV RNA, and repeat serological testing, could not be performed during the patient’s clinical course. The etiological evaluation included testing for HAV, HBV, HCV, HEV, and SARS-CoV-2. Anti-HAV IgM, anti-HCV/HCV Ag/Ab, anti-HEV IgM/IgG, and SARS-CoV-2 PCR were negative, whereas HBsAg was positive. According to the available records, the diagnostic work-up did not include investigations for other infectious etiologies (e.g., CMV, EBV, HSV, or adenovirus) or for other potential causes of pediatric acute liver failure, such as metabolic or genetic disorders, toxic exposures, immune-mediated conditions, or sepsis. Therefore, the retrospective data do not permit definitive attribution of the acute liver injury and fulminant clinical course to HBV alone.

The second patient, the boy, was initially hospitalized at “Dr. Kiro Popov” Hospital, Karlovo, on 8 April 2023 and was transferred to the Clinic of Infectious Diseases, University Hospital “St. George”, Plovdiv, on 10 April 2023, where he remained until 5 May 2023. Symptom onset was estimated to have occurred approximately two days before the initial hospitalization. Extended serological testing was carried out during the first few days after transfer to the Clinic of Infectious Diseases, approximately 5–7 days after symptom onset. HBV DNA and HCV RNA were subsequently analyzed by PCR during the same hospitalization. CMV serology was repeated before discharge.

HBV and HIV serological markers (HBsAg, total anti-HBc, anti-HBc IgM, anti-HBs, HBeAg, anti-HBe, anti-HCV/HCV Ag/Ab, and anti-HIV), together with anti-EBV IgM/IgG and anti-CMV IgM/IgG, were analyzed by chemiluminescent immunoassay on automated Maglumi X3 (Snibe Diagnostic, Shenzhen, China) and Vitros 3600 systems (Ortho Clinical Diagnostics, Rochester, NY, USA), respectively. ELISA was used to measure total anti-HDV antibodies (Dia.Pro Diagnostic Bioprobes Srl, Sesto San Giovanni, Italy) and anti-HEV IgM/IgG (EUROIMMUN Medizinische Labordiagnostika AG, Lübeck, Germany). HBV DNA was quantified by real-time PCR on an automated Bioneer system and expressed in IU/mL. HCV RNA was detected by PCR/RT-PCR using the same platform.

Serological results were interpreted as positive, negative, or borderline according to the laboratory-reported cut-off and reference values. In the second patient, the anti-HBs concentration was 5.9 mIU/mL and was reported as negative (below the protective threshold). The EBV IgG concentration was 16.8 RU/mL and fell within the laboratory-defined gray zone. CMV IgM was positive, with an index value of 6.29 and a laboratory gray-zone/cut-off range of 0.8–1.1, while CMV IgG was strongly positive at >200 RU/mL. On repeat testing before discharge, CMV IgM remained positive, with an index value of 5.0, and CMV IgG was positive at 90.7 U/mL.

In that same patient, total anti-HDV antibodies were positive; however, separate anti-HDV IgM and IgG testing was not performed. HDV RNA could not be evaluated because HDV PCR was unavailable in the hospital virology laboratory. CMV DNA PCR was likewise unavailable locally, and external molecular testing for HDV RNA and CMV DNA was not pursued because of socioeconomic constraints. Accordingly, the positive total anti-HDV result was considered to be of uncertain clinical significance and was not regarded as evidence of active HDV infection; passive transfer of maternal anti-HDV antibodies could not be excluded. Similarly, the CMV serological results were insufficient to establish clinically significant CMV disease in the absence of molecular confirmation.

The exact limits of detection and quantification for some of the molecular assays were not specified in the archived laboratory reports and therefore could not be verified retrospectively. Results were interpreted according to the validated laboratory workflow and the analytical specifications of the assays used at the time of testing.

Microbiological investigations were performed when secondary bacterial infection was suspected and included urine, blood, stool, and conjunctival secretion cultures, as well as stool testing for *Clostridioides difficile* toxins A/B when indicated. Imaging evaluation consisted of abdominal ultrasonography to assess liver size, hepatic parenchymal characteristics, biliary abnormalities, ascites, and other abdominal findings.

In addition, on 21 May 2025, approximately two years after the boy’s acute episode, late virological follow-up and an epidemiological investigation of household members were conducted. The follow-up panel included HBsAg, anti-HBc IgM, total anti-HBc, anti-HBe, HBeAg, anti-HBs, and HBV DNA. The results showed negative HBV DNA, HBsAg, anti-HBc IgM, and HBeAg; positive total anti-HBc; an anti-HBs concentration of 108.69 mIU/mL; and an anti-HBe result within the laboratory gray zone.

The two cases were compared in terms of clinical course, timing of diagnostic testing, laboratory parameters, virological findings, therapeutic management, complications, follow-up results, and disease outcomes.

The study was approved by the Ethics Committee of the Medical University, Plovdiv (Protocol No. 5/29 April 2026; Statement No. R-KNE-61/8 May 2026), and conducted in accordance with the principles of the Declaration of Helsinki. All data were anonymized to ensure confidentiality.

## 3. Results

### 3.1. Clinical Characteristics of the Two Cases

Two infants from the same family with severe HBV-associated hepatitis and markedly different clinical outcomes are presented. Table 1 summarizes the chronological sequence of clinical events, timing of diagnostic testing, management, outcomes, and follow-up in the two infants. In both infants, disease onset was acute, with clinical and laboratory evidence of severe hepatocellular injury accompanied by impaired synthetic liver function. Both were HBsAg-positive. In the boy, serological findings supported acute HBV infection, with positive total anti-HDV antibodies of uncertain clinical significance and CMV IgM/IgG positivity. HDV RNA and CMV DNA testing were not performed.

According to the neonatal discharge summaries, both infants received the birth dose of the hepatitis B vaccine within 24 h of delivery. Medical records from their general practitioner confirmed receipt of the second vaccine dose at 1 month of age. Both infants developed severe hepatitis before reaching the recommended age for the third dose: the girl at 5 months of age and the boy at 4 months of age.

Neither infant received HBIG. Maternal HBsAg and HBeAg status during either pregnancy was unknown, and no antenatal HBV screening results were available in the medical records. Routine post-vaccination serological testing had not been performed before disease onset in either child. At late virological follow-up on 21 May 2025, the surviving child had negative HBsAg, undetectable HBV DNA, negative anti-HBc IgM, positive total anti-HBc, and an anti-HBs concentration of 108.69 mIU/mL.

#### 3.1.1. Case 1: Fulminant Course with a Fatal Outcome

The first case involved E.M.K., a 5-month-old female infant, the third child in the family. She was transferred to the Clinic of Infectious Diseases at the University Hospital “St. George”, Plovdiv, in critical condition following prior hospitalization at another medical institution for suspected acute hepatitis. Markedly elevated liver enzyme levels had already been documented at the initial evaluation (ALT 683 U/L, AST 1609 U/L).

At admission and during hospitalization, the infant exhibited leukocytosis, marked cytolysis, progressive hyperbilirubinemia, and severe coagulopathy. ALT levels ranged from 623 to 613 U/L and AST from 1479 to 1073 U/L. Total bilirubin increased to 347.9 μmol/L, with direct bilirubin reaching 232.5 μmol/L. Cholestatic enzyme levels were also elevated (GGT 184–217 U/L, ALP 864–1002 U/L). The coagulation sample collected at admission on 30 April 2021 was clotted and therefore unsuitable for analysis. Given the infant’s critical condition, vitamin K, FFP, and 20% human albumin were administered immediately. The first available coagulation results, obtained on 1 May 2021 after treatment had been initiated, demonstrated profound coagulopathy (PT 7.5%, INR 5.21). Repeat testing the following day showed further deterioration (PT 6.6%, INR 5.91) despite continued administration of vitamin K and FFP. APTT was 61.5 s, TT 28.1 s, and fibrinogen ranged from 0.61 to 0.69 g/L, consistent with severe impairment of hepatic synthetic function and disseminated intravascular coagulation (DIC).

HBsAg positivity was documented, with negative markers for HAV, HCV, and HEV. Because anti-HBc IgM, HBV DNA, and follow-up serology were not available, acute HBV infection could not be definitively confirmed in this infant. Accordingly, the diagnosis is best classified as presumed HBV-associated fulminant hepatitis rather than confirmed acute HBV infection. Because the available records did not fully document a comprehensive evaluation for other infectious and non-infectious causes of pediatric acute liver failure, alternative etiologies or contributing factors could not be definitively excluded. An extended HBV serological panel was not performed during hospitalization because the positive HBsAg result became available only after the infant’s death. Hospitalization lasted from 30 April to 3 May 2021, whereas the serological results became available on 10 May 2021. As the patient was hospitalized during the COVID-19 pandemic, SARS-CoV-2 PCR testing was performed and was negative. Imaging studies revealed hepatosplenomegaly and findings consistent with cholestasis. The patient received supportive and resuscitative care, including infusion therapy, S-adenosyl-L-methionine 50 mg twice daily, lactulose 5 mL three times daily, ceftriaxone 500 mg once daily, vitamin K 1 mg once daily, furosemide 4 mg once daily, famotidine 5 mg twice daily, silymarin 10 mg three times daily, pancreatin, FFP 60 mL daily, and 20% human albumin 20 mL daily. Despite these interventions, the infant had a fulminant clinical course characterized by severe acute liver injury, hepatic encephalopathy progressing to coma, DIC, and multiorgan dysfunction, and died within three days.

#### 3.1.2. Case 2: Severe Course with a Favorable Outcome

The second case involved P.M.K., a 4-month-old male infant, the fourth child in the family, hospitalized in April 2023. The illness initially presented with fever, feeding refusal, vomiting, and diarrhea, followed by the onset of jaundice. After a brief hospitalization in another medical institution, the infant was transferred to the Clinic of Infectious Diseases at the University Hospital “St. George”, Plovdiv, due to clinical deterioration.

At admission, the infant was in severe general condition, with fever, moderate systemic toxicity, subicterus, tachydyspnea, abdominal distension, decreased peristalsis, and periorbital edema. Laboratory investigations revealed marked leukocytosis (WBC 26.7 × 10^9^/L), severe cytolysis (ALT 1506 U/L, AST 5646 U/L), hyperbilirubinemia (total bilirubin 99.4 μmol/L, direct bilirubin 64.2 μmol/L), low serum cholinesterase (2400 U/L), hypoproteinemia (45.9 g/L), hypoalbuminemia (27 g/L), elevated GGT (192 U/L), markedly increased alpha-fetoprotein (37,592.6 ng/mL), and coagulopathy (PT 18.8%, INR 2.41, APTT 46.5 s, TT 26 s, D-dimer 4.41 mg/L, fibrinogen 0.86 g/L).

Serological testing supported the diagnosis of acute HBV infection: HBsAg (+), total anti-HBc (+), anti-HBc IgM (+), and anti-HBe (+), with no protective anti-HBs titer. HBV DNA was detectable at a low level (509 IU/mL). Additional serological findings included total anti-HDV positivity and CMV IgM/IgG positivity. However, total anti-HDV positivity alone, in the absence of anti-HDV IgM subclassing and HDV RNA testing, was insufficient to establish active HDV infection. Furthermore, passive transfer of maternal anti-HDV antibodies could not be excluded. Clinically significant CMV disease likewise could not be confirmed because CMV DNA testing was not performed. Urine culture yielded extended-spectrum β-lactamase (ESBL)-producing *Escherichia coli* (10^5^ CFU/mL), necessitating initiation of meropenem therapy. Blood and stool cultures were negative. Testing for *Clostridioides difficile* toxins A/B was positive and interpreted as representing probable colonization. Imaging studies demonstrated ascites and ultrasonographic evidence of bilateral hydronephrosis/pyelectasis.

The infant received prolonged supportive, replacement, and antimicrobial treatment. Ceftriaxone 500 mg once daily was administered from 11 to 14 April 2023, followed by meropenem 100 mg three times daily from 15 to 27 April 2023 for an ESBL-producing *Escherichia coli* UTI. Before transfer, at the referring hospital on 10 April 2023 at 12:00, severe coagulopathy was documented (PT 25.7%, INR 2.28), prompting intravenous administration of 2 mg of vitamin K (Konakion). Following transfer to University Hospital “St. George”, blood samples collected at around 15:00, before local administration of FFP, demonstrated persistent coagulopathy (PT 18.8%, INR 2.41). The infant subsequently received 2.5 mg of vitamin K, 60 mL of FFP, and 30 mL of 20% human albumin on 10 April, with vitamin K continued once daily until 23 April. Serial coagulation studies showed partial improvement on 12 April (PT 28.8%, INR 1.76), followed by deterioration on 15 April (PT 10.9%, INR 3.64), and gradual normalization thereafter, with PT 75.8% and INR 1.20 on 16 April, PT 72.7% and INR 1.12 on 18–19 April, PT 84.2% and INR 1.06 on 24 April, and PT 112.5% and INR 0.85 on 30 April. FFP was administered as 60 mL on 10 April, 120 mL on 12, 13, and 17 April, 60 mL on 15 April, and 100 mL on 16 April.

The treatment regimen also included 20% human albumin (30 mL on 10 and 11 April and 40 mL daily from 12 to 14 April), furosemide (5 mg twice daily, 10 April–5 May), famotidine (5 mg once daily, 11–28 April), S-adenosyl-L-methionine (60 mg twice daily, 10–30 April), and ursodeoxycholic acid (60 mg three times daily, 20 April–5 May). Additional adjunctive therapy during hospitalization consisted of pancreatin, silymarin, vitamin C, vitamin B complex, and Saccharomyces boulardii. By day 25, the infant showed marked clinical and laboratory improvement, with resolution of severe hepatic dysfunction and coagulopathy, indicating transition to the convalescent phase. At the two-week follow-up visit, the patient was in good general condition, with normalized serum bilirubin levels and only mild residual transaminase elevation (ALT 50 U/L, AST 80 U/L).

### 3.2. Comparative Analysis of the Two Infants

Despite the shared epidemiological context, the two infants had strikingly different clinical outcomes. Both presented with severe hepatocellular injury and coagulopathy; however, the girl’s condition rapidly deteriorated, resulting in a fulminant clinical course with hepatic encephalopathy, coma, and death. In contrast, the boy had a severe but more prolonged clinical course and ultimately recovered after extended hospitalization and supportive treatment.

The cases differed mainly in the degree of coagulation impairment, timing of diagnosis, duration of hospitalization, the range of virological tests performed, and additional findings in the boy, namely total anti-HDV positivity, CMV IgM/IgG positivity, and a secondary ESBL-producing *Escherichia coli* UTI. Because active HDV infection and clinically significant CMV disease could not be confirmed by molecular testing, neither finding can be considered to have a causal or disease-modifying role in the severity of hepatitis.

### 3.3. Household HBV/HDV Cluster and Possible Transmission Scenarios

Investigation of household members found chronic HBV infection in the mother and maternal grandmother and evidence of previous HBV exposure in the eldest sibling. The mother was HBsAg-positive and HBeAg-positive, with an HBV DNA level of 87,092 IU/mL. The maternal grandmother was HBsAg-positive and anti-HDV-positive, with an HBV DNA level of 16,284 IU/mL. HDV RNA was subsequently detected in both women at levels of 411,250 copies/mL in the mother and 423,750 copies/mL in the maternal grandmother. These findings support ongoing HBV/HDV circulation within the household.

**Figure 1:**
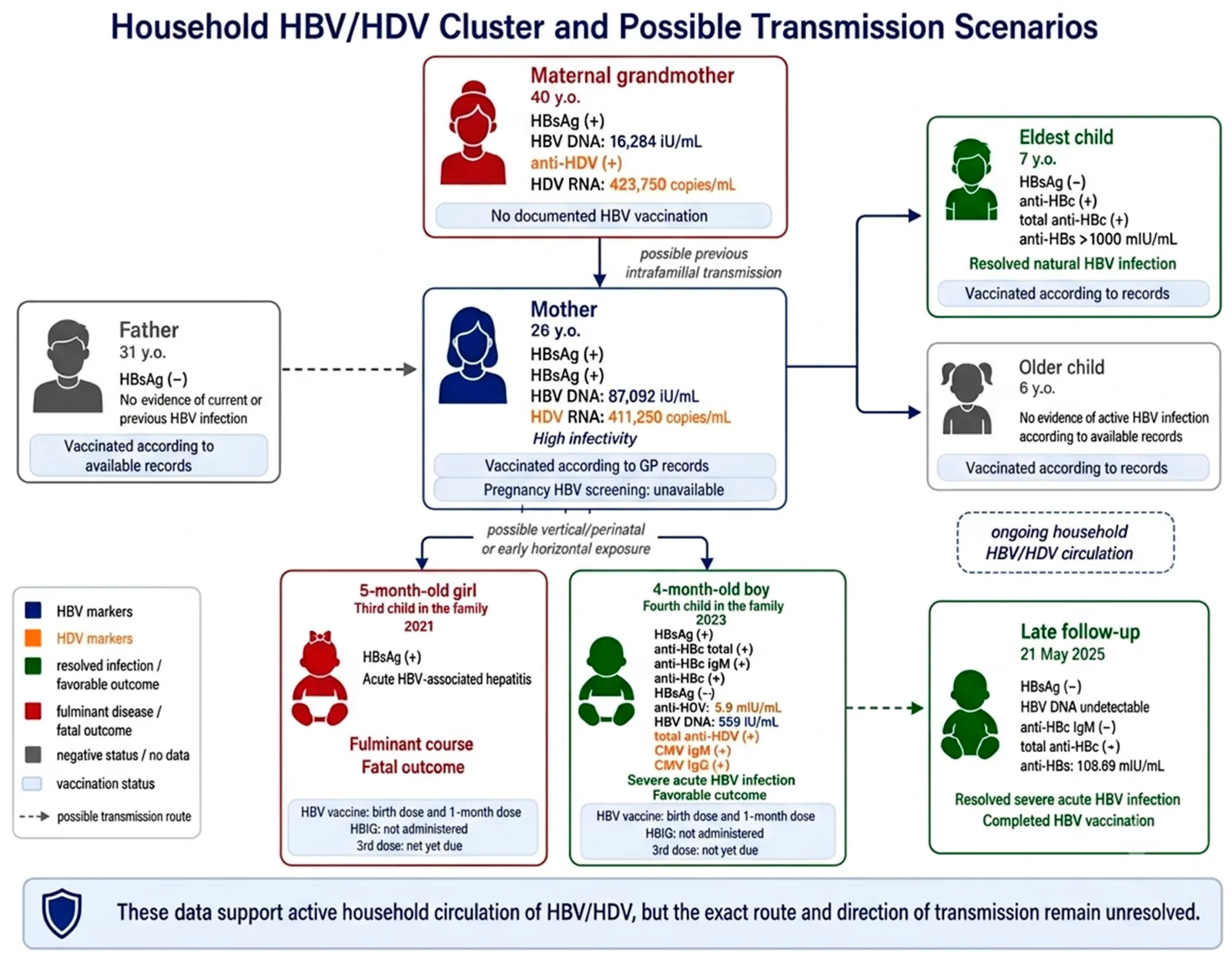
Household HBV/HDV cluster and possible transmission scenarios. The figure summarizes HBV and HDV markers, viral load data, vaccination status, and clinical outcomes among household members. Arrows indicate epidemiologically plausible exposure pathways and do not imply a proven direction of transmission. Without HBV genotyping, whole-genome sequencing, or phylogenetic comparison of viral isolates from the infants, repeated vertical or perinatal exposure, early horizontal intrafamilial transmission, and unresolved household exposure cannot be excluded. In the boy, the positive total anti-HDV antibody result is of uncertain clinical significance, as active HDV infection was not confirmed and passive transfer of maternal anti-HDV antibodies cannot be excluded.

Figure 1 provides a structured overview of the household HBV/HDV cluster, including virological findings, vaccination status, clinical outcomes, and epidemiologically plausible transmission scenarios. Table 2 summarizes individual virological findings, vaccination status, and epidemiological interpretation for each household member.

The father had no serological evidence of current or previous HBV infection. The eldest sibling had a serological profile consistent with resolved natural HBV infection, whereas the second older sibling had no evidence of active HBV infection based on the available medical records. Both infants described in this report had received the birth dose of the hepatitis B vaccine and the second dose at 1 month of age; however, neither had received HBIG. Because severe hepatitis developed before the scheduled third dose, it was never administered.

The 5-month-old girl, the third child in the family, developed presumed HBV-associated fulminant hepatitis in 2021 and experienced rapid clinical deterioration, ultimately resulting in death. The 4-month-old boy, the fourth and youngest child, developed severe acute HBV infection in 2023 and recovered after prolonged treatment, with sustained improvement during follow-up.

Although the mother and maternal grandmother were both epidemiologically plausible sources of HBV exposure, the exact source, route, timing, and direction of transmission could not be determined. No biological material from either infant was available for retrospective HBV genotyping, whole-genome sequencing, or phylogenetic comparison. Therefore, the household data should be interpreted as evidence of a familial HBV/HDV cluster rather than a defined transmission chain.

Because the two infants developed severe hepatitis in different years, several transmission scenarios remain plausible, including repeated vertical or perinatal transmission from the subsequently identified HBsAg-positive/HBeAg-positive mother, early horizontal intrafamilial transmission after birth, and unresolved household exposure in the setting of persistent HBV/HDV circulation. The available data do not allow these proposed explanations to be confirmed with certainty.

After the household-level summary, the detailed laboratory and virological characteristics of the two infants are summarized in Table 3.

## 4. Discussion

The two cases presented herein illustrate a rare but clinically significant presentation of severe HBV-associated hepatitis in infancy. Although HBV exposure in early childhood is most commonly associated with vertical or early intrafamilial transmission and subsequent chronicity, severe symptomatic liver injury, including acute liver failure, may also occur. In the girl, acute HBV infection could not be definitively confirmed because anti-HBc IgM, HBV DNA, repeat HBsAg testing, and follow-up serology were unavailable. Moreover, a comprehensive etiological evaluation for pediatric acute liver failure, including other relevant infectious etiologies, metabolic or genetic disorders, toxic exposures, immune-mediated conditions, and sepsis, was not fully documented. Therefore, her episode was classified as presumed HBV-associated fulminant hepatitis. In the boy, acute HBV infection was supported by serological and molecular findings. This clinical variability complicates prognostication and underscores the need for careful evaluation of factors that may influence the clinical course in this age group.

A central question raised by these observations is why two infants with a similar epidemiological background experienced markedly different clinical outcomes. In both cases, severe hepatocellular injury, coagulopathy, and impaired hepatic synthetic function were evident. However, the first infant rapidly deteriorated, developing severe acute liver injury with a fulminant course complicated by encephalopathy, DIC, and multiorgan failure. In contrast, the second infant experienced a severe but protracted course and ultimately recovered following intensive supportive treatment.

One possible explanation for the contrasting clinical courses is variability in the individual immune response. Fulminant hepatitis B has traditionally been attributed to immune-mediated hepatocellular injury rather than to a direct cytopathic effect of the virus. In infancy, however, the immune system is still undergoing maturation, and the balance between viral control and immunopathology may vary substantially. In the present report, differences in the immune response may have contributed to the observed clinical outcomes, although this remains speculative because immunological, genetic, cytokine, and histopathological investigations were not performed.

This interpretation is consistent with previously published observations. Dupuy et al. described a series of 14 infants aged 2–5 months with clinical presentations ranging from severe to fulminant hepatitis B; despite intensive supportive care, eight infants died. The authors emphasized that severe disease may occur even in infants who subsequently clear the virus, suggesting that the pathogenesis is not solely determined by persistent viral replication [9].

The therapeutic management of the two infants was not directly comparable. The girl had an extremely short hospitalization and experienced rapid clinical deterioration. Etiological confirmation was obtained after the fatal outcome, when the HBsAg result became available. Consequently, there was little opportunity for extended virological investigations, antiviral decision-making, or transfer for advanced liver support. In contrast, the boy’s prolonged hospitalization allowed for a more comprehensive diagnostic evaluation, pediatric gastroenterology consultation, and treatment to be adjusted according to his clinical and laboratory course. The temporal sequence of events was more clearly documented in his case. Before transfer, his INR was 2.28 at the referring hospital. Repeat coagulation testing performed after intravenous vitamin K but before FFP administration demonstrated persistent coagulopathy (INR 2.41). These laboratory data indicate that the coagulopathy criterion for pediatric acute liver failure was met, although subsequent INR values were influenced by continued treatment with vitamin K and FFP.

In the girl, treatment was necessarily supportive and resuscitative. It included infusion therapy, vitamin K, FFP, 20% human albumin, lactulose, ceftriaxone, famotidine, furosemide, S-adenosyl-L-methionine, silymarin, pancreatin, and resuscitative measures. Because etiological confirmation of HBV infection was obtained only after the fatal outcome, nucleos(t)ide analogue therapy was not initiated. In retrospect, the infant had severe acute liver injury with profound coagulopathy, hepatic encephalopathy progressing to coma, DIC, and multiorgan failure, with clinical features compatible with pediatric acute liver failure. However, because the coagulation sample collected at admission was clotted and vitamin K and FFP were initiated immediately, persistent INR elevation after vitamin K administration and before the first FFP transfusion could not be demonstrated. Nevertheless, the first available INR values remained markedly elevated despite treatment, supporting persistent severe coagulopathy. Liver transplantation was considered by the treating team; however, consultation with a transplant center or transfer to such a center was neither documented in the available medical records nor achieved because of the rapid clinical deterioration and fatal outcome within three days.

In the boy, the longer clinical course allowed a more extensive diagnostic evaluation and therapeutic adjustment. Supportive and replacement treatment included infusion therapy, vitamin K, FFP, albumin, ursodeoxycholic acid, S-adenosyl-L-methionine, furosemide, gastroprotective therapy, and symptomatic treatment, together with targeted antimicrobial therapy for an ESBL-producing *Escherichia coli* UTI. The case was discussed with a pediatric gastroenterologist, and nucleos(t)ide analogue therapy was not initiated. The rationale for this decision was not documented in the available medical records. The low HBV DNA level, HBeAg negativity, and progressive clinical and laboratory improvement may have influenced this decision, although this cannot be confirmed retrospectively. However, these factors alone should not exclude the possibility of benefit from antiviral therapy in infants with severe HBV-associated liver injury. Evidence supporting nucleos(t)ide analogue therapy for severe acute HBV infection or HBV-associated acute liver failure during infancy remains limited, although successful treatment with tenofovir has been reported in selected fulminant cases [10]. Therefore, decisions regarding antiviral therapy should be individualized and made early in consultation with a pediatric hepatologist or transplant center when severe coagulopathy, encephalopathy, or other features compatible with pediatric acute liver failure are present.

In the boy, serology was positive for total anti-HDV antibodies and CMV IgM/IgG. However, total anti-HDV positivity alone, in the absence of anti-HDV IgM subclassing or HDV RNA testing, does not confirm active HDV infection. Because the infant was 4 months old and the mother was subsequently found to have active HBV/HDV infection, passive transfer of maternal anti-HDV antibodies could not be excluded.

HDV is of particular interest because it is associated with more severe HBV-related liver disease. In this family cluster, subsequent detection of HDV RNA in the mother and maternal grandmother supports ongoing household circulation of HDV. Nevertheless, these household findings do not constitute direct evidence of active HDV infection in the infant, and the infant’s total anti-HDV antibody result alone is insufficient to conclude that HDV contributed to the severity of the clinical course.

The CMV findings should also be interpreted with caution. Positive CMV IgM together with strongly positive CMV IgG may indicate recent, congenital, or early acquired CMV infection. However, in the absence of CMV DNA PCR, viral load assessment, antigen detection, or tissue-based evidence, clinically significant CMV hepatitis cannot be established. Accordingly, CMV seropositivity may have represented an incidental finding, reflected recent exposure, or contributed to the clinical course; however, its role in the development of liver injury remains unproven.

The low HBV DNA level in the boy (509 IU/mL), despite severe hepatitis, may be explained by several non-mutually exclusive mechanisms. These include PCR testing later in the course of hospitalization, a resolving phase of acute HBV infection, immune-mediated hepatocellular injury despite low-level viremia, or a combination of these factors. Because HDV RNA testing was not performed and total anti-HDV positivity may also reflect passive maternal antibody transfer, the available data are insufficient to conclude that the low HBV DNA level resulted from HDV-mediated suppression of HBV replication.

This interpretation is consistent with the observations of Komatsu et al., who, in a case of severe acute hepatitis B in an infant born to an HBeAg-positive mother, did not identify specific HBV variants to account for the clinical course and suggested that host-related factors may be more important than viral ones in determining disease severity [11]. This inference is particularly relevant to the present study, as the two cases described herein occurred in a similar epidemiological context yet resulted in markedly different clinical outcomes.

The role of individual immunological and genetic factors should also be considered. According to Chang, age at infection, maternal HBsAg and HBeAg status, host immune status, and possibly viral strain characteristics are the principal determinants of HBV disease course in childhood [12]. In addition, differences in immune maturation, innate antiviral responses, and genetic predisposition to immunopathology may account for variability in clinical course even under similar exposure conditions [14,15]. However, these factors were not investigated in the present study, and their contribution remains hypothetical.

From a clinical perspective, the present cases underscore the importance of prompt, comprehensive diagnostic evaluation in infants presenting with severe acute hepatitis. This includes expanded virological testing for HBV, HDV, and herpesviruses, together with systematic assessment of bacterial complications. The second case further highlights the value of a multidisciplinary approach involving infectious disease specialists, gastroenterologists, nephrologists, and clinical virologists in the management of complex multifactorial clinical situations. These considerations are consistent with current public health priorities in Bulgaria, including early diagnosis, HBsAg screening during pregnancy, timely administration of the HBV vaccine at birth, active case finding, and follow-up of exposed contacts to reduce HBV transmission.

The available evidence suggests ongoing HBV circulation within the extended family, indicating a persistent risk of intrafamilial transmission. However, detailed epidemiological analysis, including HBV whole-genome sequencing and genotyping in active carriers, is beyond the scope of the present study and will be addressed in a separate manuscript.

Several limitations of the present study should be acknowledged. The report is based on two clinical cases and a retrospective analysis of medical records. In the first case, the positive HBsAg result became available only after the fatal outcome, preventing extended HBV serological profiling during hospitalization and thereby limiting precise virological characterization of the case. In addition, HBV genotyping and phylogenetic analysis were not performed, precluding a more precise assessment of transmission pathways. Another limitation is that HDV RNA testing was not performed in the boy. Consequently, his total anti-HDV positivity can only be interpreted as a serological finding of uncertain clinical significance, because active HDV infection could not be established and passive transfer of maternal anti-HDV antibodies remains a plausible explanation. Although HDV RNA was detected in the mother and maternal grandmother, these findings provide epidemiological support for possible household exposure to HDV but do not confirm active HDV infection in the infant. Immunological, genetic, and epigenetic factors that may have influenced disease outcome were also not investigated. Nevertheless, the main strength of the present manuscript lies in the detailed clinical description, the marked contrast in clinical outcomes, and the documented familial context of infection.

Taken together, these two cases illustrate the broad clinical spectrum of HBV-associated hepatitis in infancy, ranging from rapidly progressive fatal liver failure to severe but reversible disease. Several factors may have contributed to the observed variability, including viral characteristics, possible additional viral exposures, secondary bacterial infection, host response, timing of diagnostic testing, and therapeutic context. Their individual causal roles, however, cannot be determined from the available data. The present observations highlight the importance of early recognition, comprehensive diagnostic evaluation, intensive supportive care, and systematic investigation of household contacts.

## 5. Conclusions

HBV-associated hepatitis in infancy may present as a severe, potentially life-threatening condition, although such presentations are uncommon. The two infants described in this report developed severe liver injury in the context of documented HBV exposure within the same household but had distinct clinical and virological profiles. The girl had presumed HBV-associated fulminant hepatitis with a rapidly fatal course, whereas the boy had serological and molecular evidence of acute HBV infection and recovered with subsequent HBsAg clearance and undetectable HBV DNA.

The confirmed findings include HBsAg positivity in both infants, serological and molecular evidence of acute HBV infection in the boy, severe impairment of hepatic synthetic function, and the identification of a familial HBV/HDV cluster involving the mother and maternal grandmother. Follow-up of the surviving boy demonstrated HBsAg negativity, undetectable HBV DNA, and protective anti-HBs. Although the household investigation supports active HBV/HDV circulation within the family, the exact source, route, timing, and direction of transmission cannot be determined without infant HBV genotyping, whole-genome sequencing, or phylogenetic comparison.

Several factors may have influenced the divergent clinical courses, including differences in diagnostic timing, opportunity for therapeutic intervention, host immune response, the presence of total anti-HDV antibodies of uncertain clinical significance, CMV seropositivity, and secondary bacterial infection. However, the contribution of these factors remains speculative because active HDV infection and clinically significant CMV disease could not be confirmed in the surviving infant, the positive total anti-HDV antibody result may also reflect passive maternal antibody transfer, and immunological and genetic determinants were not assessed.

Taken together, these observations underscore the importance of antenatal HBV screening, timely administration of the HBV vaccine at birth, appropriate use of HBIG when indicated, prompt diagnostic evaluation of infants presenting with acute hepatitis, and systematic investigation and follow-up of household contacts in families affected by HBV infection.

## Figures and Tables

**Table 1:** Chronological comparison of the two infants with severe HBV-associated liver injury.

Stage	Child 1—Girl, 5 Months Old, Third Child in the Family, 2021, Fatal Outcome	Child 2—Boy, 4 Months Old, Fourth Child in the Family, 2023, Favorable Outcome
Symptom onset	Approximately 28 April 2021, about 2 days before admission to University Hospital “St. George”, Plovdiv	6 April 2023
First medical examination	30 April 2021—Initial evaluation by the Emergency Medical Care Center—Karlovo, followed by referral to the Clinic of Infectious Diseases because of suspected acute hepatitis and elevated transaminases	8 April 2023—Initial hospitalization at the Infectious Diseases Department, “Dr. Kiro Popov” Hospital, Karlovo
Admission/transfer to University Hospital “St. George”—Plovdiv	30 April 2021—Admission to the Clinic of Infectious Diseases, University Hospital “St. George”, Plovdiv	10 April 2023—Transfer to the Clinic of Infectious Diseases, University Hospital “St. George”, Plovdiv
Timing of initial virological samples	Immediately after admission, approximately 2 days after symptom onset	Within the first days of hospitalization at University Hospital “St. George”, approximately 5–7 days after symptom onset
Main virological findings	HBsAg-positive; anti-HAV IgM, anti-HCV/HCV Ag/Ab, and anti-HEV IgM/IgG negative	HBsAg-positive; total anti-HBc positive; anti-HBc IgM positive; anti-HBe positive; anti-HBs: 5.9 mIU/mL; total anti-HDV positive (uncertain clinical significance); CMV IgM and CMV IgG positive
Availability of results during the clinical course	The virological report was issued on 10 May 2021, after the fatal outcome	Serological results were available during hospitalization; HBV DNA and HCV RNA were tested later during the same hospitalization
Molecular testing	HBV DNA, HDV RNA, and CMV DNA were not performed	HBV DNA: 509 IU/mL; HCV RNA negative; HDV RNA and CMV DNA were not performed
Main therapeutic management	Supportive and resuscitative treatment, including vitamin K, fresh frozen plasma (FFP), albumin, ceftriaxone, lactulose, diuretic and gastroprotective therapy; detailed treatment regimens and dosages are provided in Section 3.1.1.	Prolonged supportive, replacement, and antimicrobial treatment, including vitamin K, FFP, albumin, ceftriaxone followed by meropenem, diuretic and gastroprotective therapy; detailed treatment regimens and dosages are provided in Section 3.1.2.
Clinical course	Rapid progression to severe coagulopathy, hepatic coma, DIC, and multiorgan failure	Severe disease with a prolonged course and gradual clinical and laboratory improvement
Outcome	Fatal outcome on 3 May 2021	Discharged on 5 May 2023 during the convalescent phase, with a favorable outcome
Early follow-up	Not possible because of the fatal outcome	15 May 2023—Follow-up examination after discharge, with normalization of bilirubin and mild residual elevation of transaminases
Late virological follow-up	Not applicable	21 May 2025—HBsAg-negative, HBV DNA negative, anti-HBc IgM negative, total anti-HBc positive, anti-HBs: 108.69 mIU/mL

**Table 2:** Household virological status, vaccination status, and exposure interpretation.

Household Member	Virological Findings	Vaccination/Screening Status	Interpretation
Maternal grandmother	HBsAg-positive; HBV DNA 16,284 IU/mL; anti-HDV positive; HDV RNA 423,750 copies/mL	No documented HBV vaccination	Chronic HBV infection with detectable HDV RNA; supports active HBV/HDV circulation within the household
Mother	HBsAg-positive; HBeAg-positive; HBV DNA 87,092 IU/mL; HDV RNA 411,250 copies/mL	Vaccinated according to general practitioner records; no post-vaccination serology available; antenatal HBV screening unavailable	Epidemiologically plausible source of vertical/perinatal or early horizontal exposure; cannot be proven without sequencing
Father	No serological evidence of current or previous HBV infection	Vaccinated according to available records; no post-vaccination serology available	Unlikely source of exposure based on available serology
Eldest sibling	HBsAg-negative; anti-HBe positive; total anti-HBc positive; anti-HBs > 1000 mIU/mL	Vaccinated according to records	Serological profile consistent with resolved natural HBV infection
Second older sibling	No evidence of active HBV infection according to available medical records	Vaccinated according to records	No evidence of active HBV infection in the available documentation
5-month-old girl, third child	HBsAg-positive; extended HBV serology, HBV DNA, and HDV testing not performed	HBV birth dose and second dose at 1 month; HBIG not administered; third vaccine dose was not yet due	Presumed HBV-associated fulminant hepatitis with fatal outcome; alternative etiologies could not be fully excluded; route and timing of infection cannot be determined
4-month-old boy, fourth and youngest child	HBsAg-positive; acute HBV infection supported by serology and HBV DNA; total anti-HDV positive (uncertain clinical significance); passive maternal antibody transfer cannot be excluded; CMV IgM/IgG positive; HDV RNA and CMV DNA not performed	HBV birth dose and second dose at 1 month; HBIG not administered; third vaccine dose was not yet due	Severe acute HBV infection with favorable outcome; total anti-HDV positivity does not confirm active HDV infection; clinically significant CMV disease was not molecularly confirmed

Abbreviations: HBV, hepatitis B virus; HDV, hepatitis D virus; HBIG, hepatitis B immunoglobulin; HBsAg, hepatitis B surface antigen; HBeAg, hepatitis B e antigen; anti-HBc, antibody to hepatitis B core antigen; anti-HBe, antibody to hepatitis B e antigen; anti-HBs, antibody to hepatitis B surface antigen.

**Table 3:** Laboratory and virological characteristics of the two infants.

Parameter	Reference Interval/Value Context	Girl, 5 Months Old, 2021	Boy, 4 Months Old, 2023
Leukocytes (WBC)	Reference interval 6–21 × 10^9^/L; admission/early hospitalization value	21.25 × 10^9^/L	26.7 × 10^9^/L
ALT	0–45 U/L; pre-transfer/admission value, peak value, or hospitalization dynamics	683 U/L before transfer; 623–613 U/L during hospitalization	1436 U/L on 10 April; peak recorded value 1506 U/L; 121 U/L on 5 May
AST	0–60 U/L; pre-transfer/admission value, peak value, or hospitalization dynamics	1609 U/L before transfer; 1479–1073 U/L during hospitalization	4806 U/L on 10 April; peak recorded value 5646 U/L; 155 U/L on 5 May
Total bilirubin	3.4–21 μmol/L; peak value or hospitalization dynamics	Up to 347.9 μmol/L	95.9 μmol/L on 10 April; peak 101.4 μmol/L on 24 April; 33.3 μmol/L on 5 May
Direct bilirubin	0.8–8.5 μmol/L; peak value or hospitalization dynamics	Up to 232.5 μmol/L	Peak recorded value 64.2 μmol/L; 62.3 μmol/L on 24 April; 16.6 μmol/L on 5 May
GGT	0–55 U/L; hospitalization range or available values	184–217 U/L	211 U/L on 10 April; range 134–235 U/L during hospitalization; 229 U/L on 5 May
ALP	42–362 U/L	864–1002 U/L	304 U/L on 16 April
LDH	230–975 U/L	n.a.	2963 U/L on 10 April; 1791 U/L on 12 April; 751 U/L on 16 April
Albumin	35–52 g/L; nadir or hospitalization dynamics	n.a.	28.0 g/L on 10 April; nadir 27 g/L in the medical records; 48.0 g/L on 16 April
Total protein	57–80 g/L	n.a.	47.9 g/L on 10 April; 57.3 g/L on 12 April; 68.8 g/L on 16 April
Serum cholinesterase	3600–11,500 U/L	n.a.	2400 U/L in the medical records; 4430 U/L on 16 April
Alpha-fetoprotein	0–9 ng/mL according to the reporting laboratory	n.a.	37,592.6 ng/mL on 16 April
PT	70–120%; available value or hospitalization dynamics	Coagulation sample at admission was clotted/unsuitable for analysis; 7.5% on 1 May; 6.6% on 2 May	18.8% early in hospitalization; nadir 10.9% on 15 April; 84.2% on 24 April
INR	No separate reference interval provided in the laboratory report	5.21 on 1 May; 5.91 on 2 May, after initiation of vitamin K and FFP	2.41 early in hospitalization; peak 3.64 on 15 April; 1.06 on 24 April
APTT	24–35 s	61.5 s	46.5 s early in hospitalization; peak 57.8 s on 15 April; 32.6 s on 24 April
TT	14–21 s	28.1 s	26 s early in hospitalization; 24.2 s on 15 April; 18.4 s on 24 April
D-dimer	0.0–0.50 mg/L; dynamic change rather than range	1.61 → 0.88 mg/L	4.41 mg/L early in hospitalization; peak 13.88 mg/L on 12 April; 0.53 mg/L on 24 April
Fibrinogen	2.0–4.5 g/L; nadir or hospitalization dynamics	0.61–0.69 g/L	0.86 g/L early in hospitalization; nadir 0.58 g/L on 12 April; 2.07 g/L on 24 April
HBsAg	Serological interpretation	Positive	Positive
Total anti-HBc	Serological interpretation	Not performed	Positive
anti-HBc IgM	Serological interpretation	Not performed	Positive
HBeAg	Serological interpretation	Not performed	Negative
anti-HBe	Serological interpretation	Not performed	Positive
anti-HBs	Protective threshold ≥ 10 mIU/mL	Not performed	5.9 mIU/mL, below protective threshold
HBV DNA	Molecular testing	Not performed	509 IU/mL
HDV markers	Serology/molecular testing	Not performed	Total anti-HDV positive (uncertain clinical significance); HDV RNA not performed; passive maternal antibody transfer cannot be excluded
CMV markers	Serology/molecular testing	Not performed	CMV IgM positive; CMV IgG positive; CMV DNA not performed
Other viral markers	Serology/molecular testing	anti-HAV IgM negative; anti-HCV/HCV Ag/Ab negative; anti-HEV IgM/IgG negative	anti-HAV IgM negative; anti-HCV/HCV Ag/Ab negative; HCV RNA negative; anti-HIV negative; EBV IgM negative; EBV IgG gray-zone; anti-HEV IgM/IgG negative
Secondary bacterial infection	Microbiological testing	Not demonstrated	ESBL-producing *Escherichia coli* 10^5^ CFU/mL in urine
*Clostridioides difficile* toxins A/B	Stool toxin testing	Not performed/not documented	Positive, interpreted as probable colonization
Imaging findings	Abdominal ultrasonography	Hepatosplenomegaly; cholestasis	Ascites; bilateral hydronephrosis/pyelectasis
Main complications	Clinical course	Severe acute liver injury with profound coagulopathy; encephalopathy/hepatic coma; DIC; multiorgan failure; clinical features compatible with pediatric acute liver failure	Coagulopathy criterion for pediatric acute liver failure met; coagulopathy; edema-ascites syndrome; secondary UTI

Table note: Values are presented as admission values, peak or nadir values, hospitalization ranges, or dynamic changes, as specified in the table. For the girl, selected pre-transfer values are included where clinically relevant. Reference intervals from the boy’s laboratory reports were used for comparison because both infants were of similar age and were tested using the same hospital laboratory system. For some parameters, including INR, no reference interval was provided. Abbreviations: ALT, alanine aminotransferase; AST, aspartate aminotransferase; GGT, gamma-glutamyl transferase; ALP, alkaline phosphatase; LDH, lactate dehydrogenase; PT, prothrombin time; INR, international normalized ratio; APTT, activated partial thromboplastin time; TT, thrombin time; DIC, disseminated intravascular coagulation; UTI, urinary tract infection; HBV, hepatitis B virus; HDV, hepatitis D virus; CMV, cytomegalovirus; HBsAg, hepatitis B surface antigen; HBeAg, hepatitis B e antigen; anti-HBc, antibody to hepatitis B core antigen; anti-HBe, antibody to hepatitis B e antigen; anti-HBs, antibody to hepatitis B surface antigen; ESBL, extended-spectrum β-lactamase; n.a., not available.

## Data Availability

The data supporting the findings of this study are available from the corresponding author upon reasonable request. The data are not publicly available due to privacy and ethical restrictions related to the clinical nature of the study and the protection of patient confidentiality.
